# Care at home for remdesivir treatment of COVID-19: a survey study of patient and physician experiences

**DOI:** 10.1186/s12879-025-11737-1

**Published:** 2025-10-23

**Authors:** Julia Nguyen, Rulin Hechter, Deborah Ling-Grant, Janis Yao, Cecilia Portugal, Albert Bai, Raul Calderon, William Towner

**Affiliations:** 1 Kaiser Permanente Southern California Home Infusion Pharmacy,, Panorama City Medical Center, 13652 Cantara Street, Bldg. 5, LL, Rm L21, Panorama City, CA 91402 USA; 2 Kaiser Permanente Department of Research and Evaluation, Southern California Permanente Medical Group, Pasadena, CA USA; 3 Department of Health Systems Science, Kaiser Permanente Bernard J. Tyson School of Medicine, Pasadena, CA USA; 4 Kaiser Permanente Southern California Infectious Diseases, Los Angeles Medical Center, Los Angeles, CA 90027 USA

**Keywords:** COVID-19, Remdesivir, Hospital in the home, Telehealth

## Abstract

**Background:**

During the COVID-19 pandemic, remdesivir (RDV) treatment and advanced medical care at home (AMCAH) were pivotal implementations for delivery of comprehensive outpatient services.

**Methods:**

A retrospective survey study was conducted to assess patient and physician experiences of RDV treatment. The online/telephone survey questionnaire was developed based on components of the Health Belief Model, Hospital Consumer Assessment of Healthcare Providers (HCAHPS), and physician change management theory. Patients ≥ 18 years of age (*N* = 1681) who received a three- or five-day RDV treatment regimen at home and prescribing physicians (*N* = 406) from December 15, 2020-August 31, 2022, in a large integrated non-profit health system were eligible. A cross-sectional analysis was performed to assess the associations of perceptions about value with patient adherence to 100% of prescribed doses, physician adoption of treatment guidelines, and patients’ healthcare utilization. Reduction in COVID-19 related readmission within 30 days of entry into treatment program was also measured. Descriptive statistics were used to describe categorical responses.

**Results:**

Of survey results from 539 patient respondents (response rate: 32%, mean age: 58.4 years (SD 16.2), 55.1% male), race/ethnicity was predominantly Hispanic (48.4%), followed by White (32.3%), and Black (12.6%). Adherence was 87% of patients having completed 100% of RDV doses at home. Patients (79.9%) reporting being “*very satisfied*” with medical follow-up were less frequently readmitted within 30 days (*p* < 0.01). Of 140 physician respondents (response rate: 34%, mean age: 46.9 years (SD 8.4), 58% male), race/ethnicity was predominantly Asian (66%), followed by White (23%), and Hispanic (7.1%). Prescribing was associated with feeling knowledgeable about RDV (*p* < 0.01) and perceived effectiveness for reducing community transmission (*p* = 0.03).

**Conclusions:**

In racially diverse samples, patients and physicians responded favorably towards AMCAH integrating RDV for treatment of COVID-19. Easy to understand communication and hospital readmission were found to most shape patient satisfaction. AMCAH during the pandemic demonstrated that acute care needs of patients can be safely met at home. These findings inform future design of health care delivery and support expansion for treatment of other acute illnesses.

**Key points:**

**Question:**

What were patient and physician responses to advanced medical care at home integrating RDV for treatment of COVID-19?

**Findings:**

In this survey study, majority of eligible patients (87%) completed 100% home RDV. Patients reported physicians most influenced their decision to accept treatment (70.7%) and high satisfaction with telehealth (95.8%). Patients who reported being “*very satisfied*” with medical follow-up (81.8%) were less frequently readmitted within 30 days (p<0.01). Physician prescribing of RDV was associated with knowledge (p<0.01) and perceived effectiveness for reducing community transmission (p=0.03). Most physicians reported confidence in nurse capacity (88.2%) and pharmacy services (96.3%) to support care at home.

**Supplementary Information:**

The online version contains supplementary material available at 10.1186/s12879-025-11737-1.

## Introduction

Advanced medical care at home (AMCAH) is a model of care that provides hospital level care at home in place of the traditional hospital setting [1]. This model has been associated with admission avoidance, shorter hospital stays, lower re-admission rate, and subsequent lower costs of care. Reimbursement rates, however, have historically hampered widespread uptake at the institutional level [2, 3].

Amid rising COVID-19 hospitalizations and repeated surges, the Centers for Medicare & Medicaid Services (CMS) issued a “Hospital without Walls” waiver that provided full hospital fee-for-service diagnosis-related group payment [4–6]. This notably catalyzed implementation of care at home programs encompassing 24 h/7 days per week patient care through home visits, telehealth encounters, and remote patient monitoring(RPM) thus offering an opportunity to better evaluate the practice [7–10]. 

Implementation of this alternative model of care hinged upon physicians who display high professional autonomy as drivers of innovation and patient acceptance as end users of health services [11, 12] Reported barriers for physician referral of patients to AMCAH have included underdeveloped workflows and difficulty screening appropriate patients [13]. Little was known how physicians would respond to rapid scale up of care at home integrating an intravenous antiviral, remdesivir (RDV), in such an urgent context [14]. As importantly, patient intent to receive and willingness to pay for such services were also not well understood and hypothesized to be similar to vaccine hesitancy [15]. 

In this survey study, we sought to better understand patient and physician perceptions of care at home with RDV for treatment of COVID-19. Our primary objectives were to (1) assess patient satisfaction (2) measure composite COVID-19 related hospital readmission and adherence defined as taking 100% of prescribed RDV doses, and (3) assess prescribing physician confidence associated with adoption of treatment guidelines for home RDV.

## Methods

### Study design & setting

We performed a retrospective study using cross-sectional electronic/telephone surveys of patients ≥ 18 years of age who received home RDV and prescribing physicians from December 15, 2020-August 31, 2022, combined with electronic medical record (EMR) data. It was conducted at a non-profit integrated health care system with fifteen medical centers that provides comprehensive preventive and medical care to approximately 4.7 million members who are demographically similar to the diverse socioeconomic, ethnic California population at large [16]. 

Data on healthcare utilization visits and readmission from EMR including outside of network utilization were used to confirm eligibility and determine adverse effects and adherence to RDV. Comorbid conditions including diabetes mellitus, hypertension, obesity (BMI >30), active malignancy (defined as receiving treatment within 180 days prior to index date), HIV/AIDS, use of immunosuppressant medication (including prednisone at ≥ 5 mg a day ≥ 14 days prior to index date), and initial oxygen saturation (O2sat) on room air when presenting for first RDV treatment, (O2sat >94% or O2sat < 94%), COVAS (comorbidity, obesity/BMI, vital signs, age, and sex) risk score were captured along with outcomes data through structured administrative and clinical databases and EpicCare-based EMR [17–24]. The Kaiser Permanente Southern California Institutional Review Board reviewed and approved all study activities with a waiver of written informed consent. All methods were conducted in accordance with relevant guidelines and regulations in accordance with the Declaration of Helsinki.

### Study population

*Inclusion criteria* were patients ≥ 18 years who were considered for hospital admission with (1) COVID-19 ICD-10 diagnosis U07.1 confirmed with a positive severe acute respiratory syndrome coronavirus 2 (SARS-CoV-2) polymerase chain reaction ≥ 10 days (to account for limited availability of point-of-care testing and delayed molecular assay results at the onset of the pandemic) and (2) who went on to receive care at home with RDV. RDV treatment duration was three days (in nonhospitalized patients with mild to moderate COVID-19 who were at high risk of progressing to severe disease) or five days (in initially hospitalized patients up to a total duration of 10 days for non-improvement) within 7 days of symptom onset consistent with COVID-19. Hospital readmission was defined as an admission to a hospital within 30 days of a discharge from the same or another hospital.

*Exclusion criteria* were concurrent administration of concurrent Janus kinase inhibitor (barcitinib), monoclonal antibody (bamlanivimab plus etesevimab, casirivimab plus imdevimab, sotrovimab, bebtelovimab, tixagevimab plus cilgavimab, tocilizumab) and > 60-day gap in continuous coverage during the study period May 1, 2020 -August 31, 2022. Patients were censored at discontinuation of insurance coverage or at the end of the study period, whichever occurred first. This period represented major hospital surge conditions in Southern California associated with the emergence of variants of concern (Alpha, Beta, Gamma Dec 29, 2020, Delta June 15, 2021, and Omicron Nov 26, 2021) first identified by the Centers for Disease Control (CDC).

### Survey tools and procedures

From the study population, all surviving eligible current health plan members and employed prescribing physicians were invited to participate via the REDCap electronic survey system and followed up by the recruitment team via reminder phone calls/text messages/emails. Individual sociodemographic characteristics were self-reported. Age, gender, race, and ethnicity were collected to confirm representativeness. All questions were offered in English and Spanish. Respondents could opt to skip questions. Pre-testing of survey questions with twenty patients and twenty providers was undertaken to assess appropriate reading level and understanding.

### Patient survey measures

Ordinal scale and binary questions from previously published surveys assessing predictors of intent to receive the COVID-19 vaccine were modified to assess demographic, health status, COVID-19 experience, willingness to pay, and intention to receive RDV based on Health Belief Model (HBM) constructs: perceived severity, perceived susceptibility, perceived benefits, perceived barriers, cues to action, and self-efficacy [25–27]. Modified validated patient satisfaction questions derived from the Hospital Consumer Assessment of Healthcare Providers and Systems (HCAHPS) were also integrated to measure patient satisfaction [28]. The HCAHPS survey is a standardized approach for measuring patient experience of care and is a critical component of U.S. federal, state, and private value-based programs and plan accreditation [29, 30] Additional questions derived from the Global Adherence Scale for Acute Conditions (GASAC) were used to assess ability to follow home isolation guidelines, ability to have transportation to and from a medical center, ability to require minimal assistance with activities of daily living, and presence of social support [31]. Health literacy was assessed using an abbreviated validated version of the Short Test of Functional Health Literacy in Adults (STOFHLA) tool [32, 33] Medication adherence threshold was defined as taking 100% of prescribed doses and at least 80% follow-up at the end of the recommended treatment period for short‐term treatments [34]. Adverse events were defined as cardiac arrest, elevated serum creatinine, elevated liver function tests >3 times upper normal limit, or hypersensitivity reaction leading to discontinuation.

### Physician survey measures

Ordinal scale questions were developed based on the Unified Theory of Acceptance and Use of Technology (UTAUT) model as a proxy for acceptability in implementation. Performance expectance, effort expectancy, social influence, facilitating conditions, and physician acceptance were assessed with questions measuring CM-compatibility; OB-observability; JR-job relevance; PD-personal demographics; PE-personal experience; INV-internal environment; EXV- external environment, BI-behavioral intention, and AT-attitude toward implementation [35–39]. Additional questions identifying level of involvement with organization planning and perception of implementation climate were developed to assess peer support, facility resources, and health status [40, 41].

### Statistical analysis

Results of patient survey and physician survey data were summarized as median and interquartile range (IQR) for continuous variables and as frequency (percentage) for categorical variables. Quantitative responses were reported as a Top 2 Box score (T2B) as a way of summarizing the highest two positive responses from the Likert scale survey questions. For patient survey data, the relationship between each question and the RDV adherence groups, patient readmission status, and adverse event status were evaluated. The Wilcox rank sum test and Chi square test were applied to basic demographic variables to assess nonresponse bias. Physician survey data were compared among different physician adoption levels of treatment guidelines. The Chi-square test or Fisher exact test was applied for categorical variables, and the Wilcoxon rank sum test was used for continuous variables to detect relationships. All analyses were two-sided and performed using SAS Enterprise Guide 8.2 (Cary, NC). P values of < 0.05 were considered statistically significant.

## Results

### Patient survey results

**Fig. 1:**
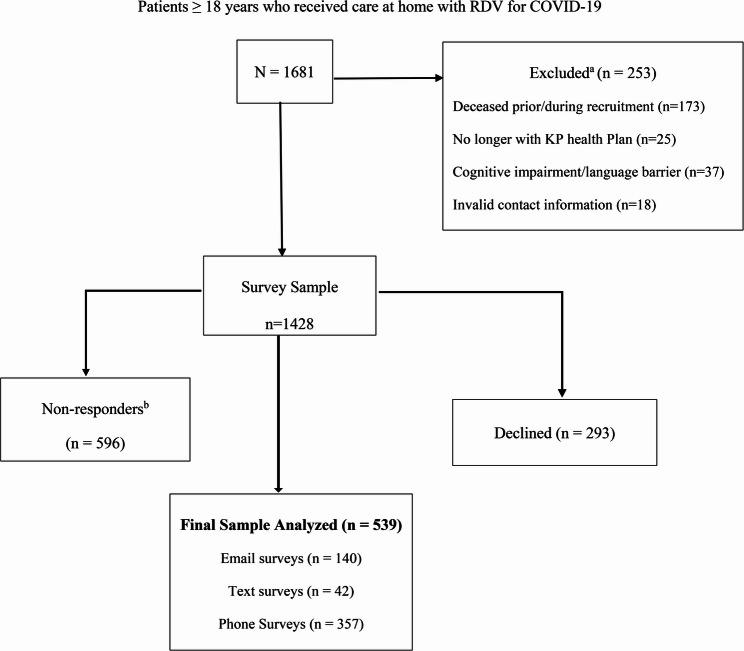
Patient survey recruitment consort flow diagram

A total of 1681 patients were eligible for survey. (Fig. 1. Patient Survey Recruitment Consort Diagram) COVID-19 vaccination rate defined as receiving ≥ 1 dose was 35.3% in respondents. Respondents were similar to non-respondents on all demographic factors except for age where median age (IQR) 59.0 years (45.0–71.0) was older than non-respondents 55.0 years (41.0–70.0), (*p* < 0.01). (Table 1. Demographic Comparison of Respondents) For respondents, there was a predominance of Hispanic race/ethnicity 48.4%. T2B score for education level (71.3%) was comprised of some college or technical school (40.8%) and bachelor’s degree or higher (30.6%). Preferred language was English 89.1%. T2B score for overall health (63.7%) was comprised of fair (26.9%) and good (36.8%). Physicians were rated as the most influencing factor 70.7% for accepting treatment for COVID-19. Amongst respondents, the frequency of hospital readmission within 30 days of treatment was 11.3% (*n* = 60) and 32.9% (*n* = 166) respectively.

**Table 1:** Demographic Comparison of Survey Nonrespondents and Respondents

	Nonrespondent/Declined(*N*=889)	Respondent(*N*=539)	Total(*N*=1428)	*P*-value
Age				0.00251
Mean (SD)	55.7 (17.57)	58.4 (16.24)	56.7 (17.13)	
Median (IQR)	55.0 (41.0, 70.0)	59.0 (45.0, 71.0)	56.0 (43.0, 70.0)	
Range	18.0, 97.0	18.0, 96.0	18.0, 97.0	
Race				0.07312
1.Asian/PI	77 (8.7%)	29 (5.4%)	106 (7.4%)	
2.Black	93 (10.5%)	68 (12.6%)	161 (11.3%)	
3.Hispanic	446 (50.2%)	261 (48.4%)	707 (49.5%)	
4.White	256 (28.8%)	174 (32.3%)	430 (30.1%)	
5.Other	17 (1.9%)	7 (1.3%)	24 (1.7%)	
Gender, *n* (%)				0.42632
F	380 (42.7%)	242 (44.9%)	622 (43.6%)	
M	509 (57.3%)	297 (55.1%)	806 (56.4%)	

^1^Wilcoxon rank sum *p*-value

^2^Chi-Square *p*-value

Adherence was found in 87% of patients having completed 100% of RDV doses and was unaffected by race/ethnicity, age, and gender. Adherent respondents vs. non-adherent respondents were more likely to have caregiver support either by having someone to help take care of daily needs “*all of the time*” 69% vs. 62.3% or helping with transportation to the doctor if needed 77% vs. 62.3%, (*p* < 0.05). Adherent respondents were more likely to report feeling “*much better”* after treatment with RDV 48.5% vs. non-adherent respondents 32.8%, (p = 0.01). Adherent respondents reported more favorably vs. nonadherent respondents to variant questions related to nursing care, (p < 0.05).

The median age of readmitted respondents versus non-readmitted was 66 years vs. 58 years, (*p* < 0.01). (Table 2. Summary of Patient Survey Responses**)** Readmitted respondents differed significantly compared to those not readmitted in three domains: lower health literacy, less positive perceptions about RDV intervention, and lower patient satisfaction with medical care, nurses, and physicians. Readmitted respondents had difficulty with understanding written information about medical conditions, 50% compared to those who were not readmitted 33%, (*p* < 0.05). Readmitted respondents reported lower perceived effectiveness of RDV related to decreasing duration of infection 56.1% vs. 68.9%, (*p* = 0.02) or improving recovery 50.9% vs. 70.6%, (*p* < 0.01). T2B score for perceiving RDV benefit outweighing potential risks was correspondingly lower in readmitted respondents 55.0% vs. 69.9%, (*p* = 0.03). Readmitted respondents reported lower patient satisfaction with nurses providing care at home 87.7% vs. 95.8%, (*p* < 0.01) and understandable nurse communication 88.1% vs. 92.4%, (*p* < 0.01). Similarly, re-admitted respondents rated lower satisfaction with medical follow-up, 54.5% “*very satisfied”* versus 81.8% in non-readmitted respondents, (p < 0.01). Readmitted respondents rated physicians treating them with courtesy and respect 86.0% vs. 92.5%, (p < 0.01). The overall proportion of positive responses is summarized. (Fig. 3. Proportion of Positive Responses to Health Belief Model Constructs for Care at Home)

Respondents who experienced an adverse event differed from the non-readmitted comparator in four categories:1) higher perceived benefit, 2) higher perceived susceptibility, 3) decreased caregiver support, and 4) decreased willingness to pay. They reported perceiving RDV as decreasing duration of infection 72.9%, (*p* < 0.05) and they reported that hospital staff described side effects in a way they could understand 49.4%, (*p* < 0.05). Readmitted respondents who experienced an adverse event also had less caregiver support 77.4% vs. 86.2%, (*p* < 0.05). Re-admitted respondents also were not willing to cost share RDV treatment (34.4%) and when willing, only up to a maximum of $50 per dose (45.6%), (*p* < 0.01).

### Physician survey results

**Fig. 2:**
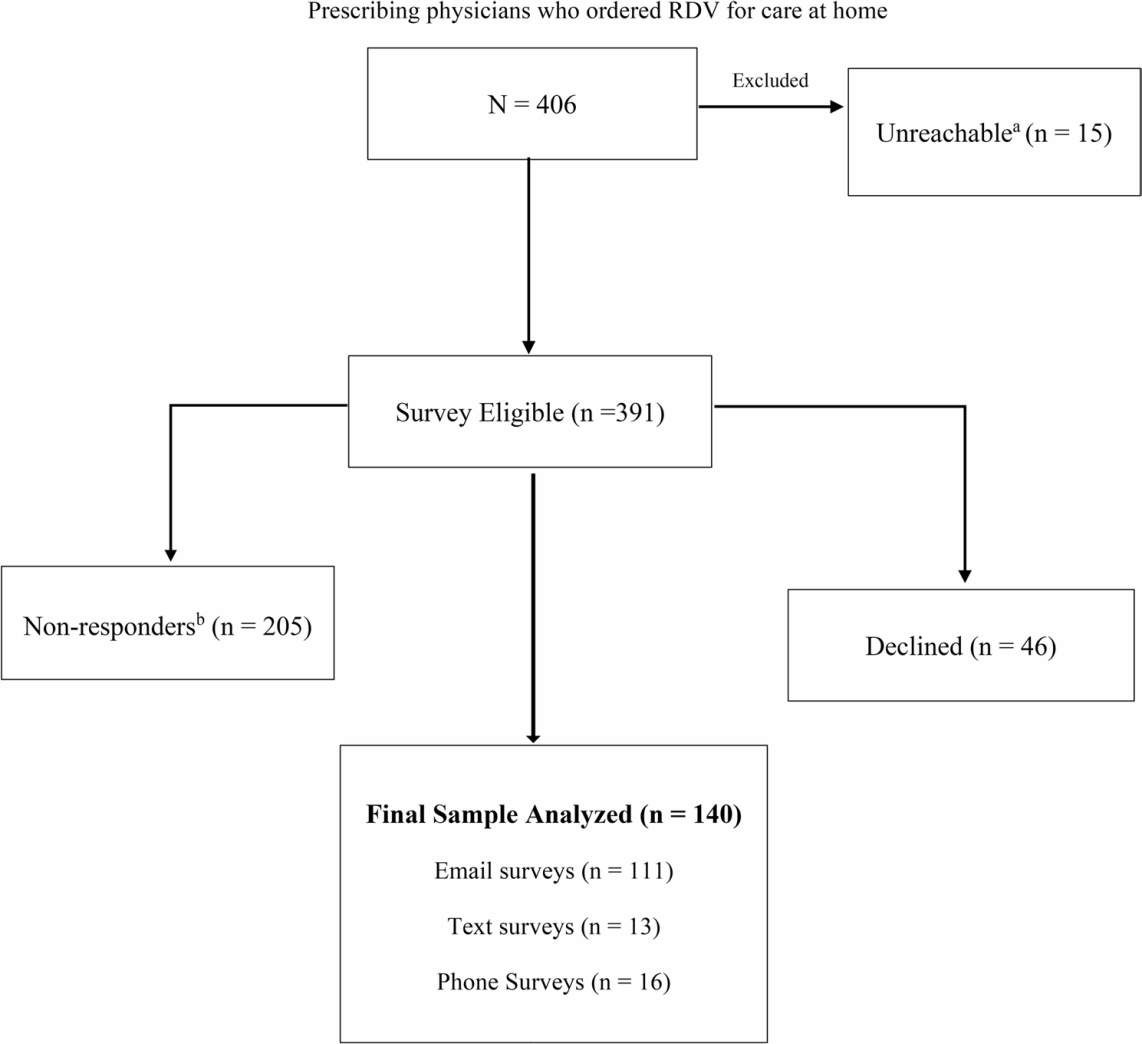
Provider survey recruitment: consort flow diagram

A total of 406 physicians were eligible for survey as depicted in Fig. 2. Provider Survey Recruitment Consort Diagram. Respondents were hospitalists (67.4%) and emergency medicine (13.0%) with ≤ 10 years of experience (48.6%). Health compared to the past year was rated “*about the same”* overall (51.8%), physical health (46.8%), mental health (50.4%) and anxiety and stress levels (46.4%). They reported care at home with RDV relieved utilization of hospital resources (93.5%). Confidence in allied healthcare partners was for pharmacy services (96.3%), support for telehealth (84.6%), care at home nursing (87%), off-site lab/phlebotomy (77.8%), and durable medical equipment supplier (77.4%). Respondents identified labor shortage (41.3%) as the highest priority to invest to overcome barriers in another medical crisis. (Table 3. Summary of Physician Survey Responses)

Among prescribing physicians, respondent strength of agreement differed significantly on questions associated with three domains: behavioral intention, attitude, and observability for ordering new or unfamiliar medications under emergency use authorization (EUA). Differences amongst personal demographics, compatibility, and external environment were not found to be significant.

#### Behavioral intention

**Fig. 3:**
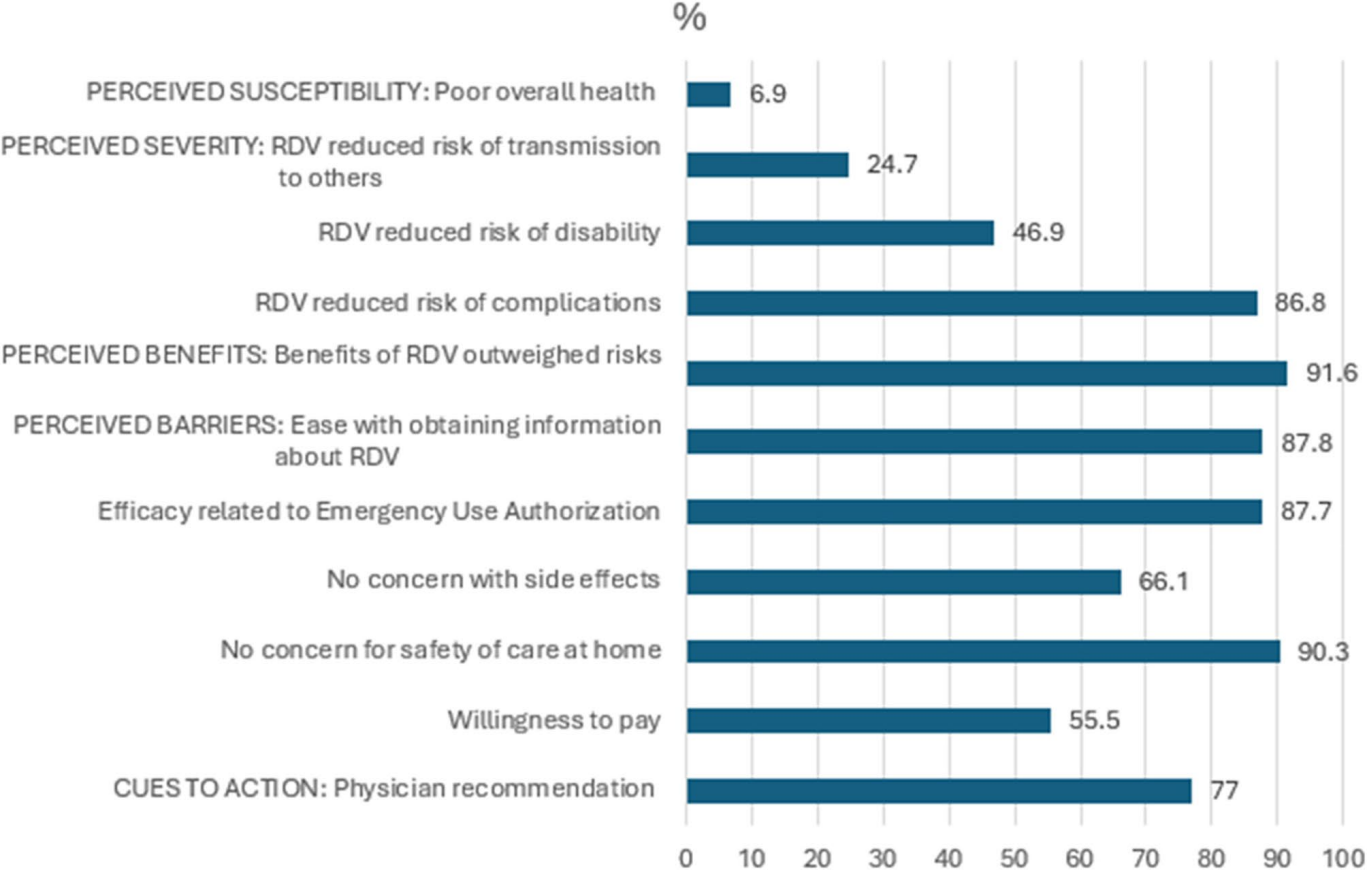
Proportion of positive responses to health belief model constructs for care at home

For self-efficacy, prescribing physicians who reported “*strongly agree*” to feeling knowledgeable about RDV (81.3%) and comfort with off-label use of COVID-19 treatment based on limited clinical data (56.3%), (*p* < 0.02) were associated with “*strongly agree*” with ordering new or unfamiliar medications under EUA. While physicians perceived that providing RDV reduced community transmission reported “*agree/strongly agree”* 72.2% vs. “*disagree*” 26.7%, (*p* = 0.03), perception of effectiveness differed during different waves. Respondents were divided in strength of agreement at the onset of the pandemic Dec 2020-June 2021 associated with alpha, beta, gamma variants reporting “*confident*” 40.9%, “*somewhat confident”* 38.7%, “*not confident*” 11.7%, and “*very confident*” 8.8%, (p = 0.02). No significant difference in strength of confidence was identified in latter periods associated with delta and omicron variants. Figure 3

#### Attitude

Amongst personality traits of agreeability, extraversion, openness, being conscientious rated on a scale of 1–5 with five being the highest, being “*always*” open 52.9%, (*p* = 0.02) was most associated with ordering new or unfamiliar medication under EUA.

#### Observability

Physicians who expressed peer influence in decision making with adherence to COVID-19 treatment guidelines differed with strength of agreement where *“agree/strongly agree*” (62.3%) prevailed, (p<0.01).

## Discussion

AMCAH requires patient and physician engagment [41–43]. Our survey results found that with physician guidance, patients were receptive to care at home. High ratings of medical follow up, telehealth encounters, and low adverse event frequency were consistent with previously reported literature [44–46]. Frequency of readmission in this cohort 11.3% was lower than reported state-wide pre-pandemic admission rate 14.9% in California [46]. Combined with favorable physician responses towards AMCAH, these findings in a racially diverse sample support expansion to include treatment of other acute illnesses that do not require emergency critical care or procedures.

Of additional interest are patient satisfaction findings associated with hospital re-admission. Despite social screening and clinical stratification of patients, health literacy and level of caregiver support differed significantly in readmitted respondents. Readmission significantly affected patient satisfaction with care with reported lower ratings of communication, care provided by nurses and physicians, perception of RDV effectiveness, and willingness to cost-share for RDV treatment. Repeated patient education at all points of patient contact may be a high yield strategy to reframe expectations of an intervention and prospectively facilitate high patient satisfaction [47]. 

Although physicians were identified as the factor most influencing patient decision making, we were unable to corroborate reports of decreased trust in physicians and health related behaviors during the pandemic [48]. Furthermore, the low COVID-19 vaccination rate in patients receiving RDV at home was much lower than overall 81% reported within our regional health system and 67% nation-wide[49, 50]. Implications of these results underscore vaccination efforts as a critical preventive intervention even with the availability of treatments to improve outcomes and decrease utilization of acute care services [51–52].

Survey results provided key insights about behavioral intention, attitudes, and observability among prescribing physicians in the setting of high confidence with technology, nursing, and pharmacy services. Although physicians were comfortable with off-label uses of treatments for COVID-19 and RDV, knowledge about treatments appeared to most influence their sense of self-efficacy. Hence, it was not surprising to find perception of RDV effectiveness was most divided at the onset of the pandemic that could be attributed to conflicting evidence-based guidance. Despite differences in levels of openness and agreement as to the extent of peer influence, a “learning curve” was observed. Emergence of less virulent strains and data feedback reinforcing practice appears to have progressively strengthened a trend towards cohesive aligned prescribing. This highlights physician autonomy in driving implementation in the context of organizational policies and practice [53]. 

### Limitations

Recall bias may have affected survey response since participants were required to recall experiences as far back as three years. Finding that overall, physical, and mental health of physicians was unchanged pre- and post-pandemic was intriguing. While suggestive of resilience and a hypothetical insulative practice buffer provided by an integrated health system, physician recovery from pandemic induced stress may have affected recall.

Given longitudinal observational data of the RDV cohort without a comparison cohort, we were unable to match and compare baseline covariates which may have resulted in a biased treatment effect. Likewise, we were unable to adjust for potential confounding arising from time varying treatment effectiveness for different SARS-CoV-2 variants during the study period. We attempted to address this potential confounder by surveying physician perception of effectiveness during different variant time periods.

Lastly, the use of proxy variables to describe factors that influenced implementation of AMCAH in an integrated health care system may limit extrapolation of findings to different practice settings. Selection bias may have also skewed survey results reporting patient receptivity to RPM technology and telehealth. Value based outcomes where value was defined as quality over cost were not assessed and represent a future area of investigation.

## Conclusion

Initially intended for use in a healthcare setting capable of providing acute care comparable to inpatient hospital care, early availability of RDV positioned it well as treatment for integration into care at home. In racially diverse samples, patients and physicians responded favorably to this model of care for management of COVID-19. Easy to understand communication and hospital readmission were found to most shape patient satisfaction. Use of a standard survey tool contributed to real world application of research to incentivize quality care and produce comparable transparent data on patients’ perspectives within healthcare. AMCAH during the pandemic provided proof of concept that acute care needs of patients can be safely met at home. Infection control may further be enhanced with appropriate isolation guidelines. These findings inform future design of health care delivery and support expansion for treatment of other acute illnesses.

## Supplementary Information

Supplementary Material 1: Table 2. Summary of Patient Survey Responses: Hospital Re-admission Indicator

Supplementary Material 2: Table 3. Summary of Physician Survey Responses

## Data Availability

The data used for this study contain protected health information; thus, individual level data may not be made publicly available due to the Institutional Review Board, business, and privacy concerns. Data are available for researchers who meet the criteria for access to confidential data. Please contact the KPSC Institutional Review Board ([kpsc.irb@kp.org](mailto: kpsc.irb@kp.org)) for more information.

## Abbreviations

AMCAH: Advanced Medical Care at Home
BMI: Body Mass Index
CMS: Centers for Medicare & Medicaid Services
COVAS score: comorbidities, obesity, vital signs, age, sex
COVID-19: Coronavirus Disease of 2019
EMR: Electronic Medical Record
EUA: Emergency Use Authorization
GASAC: Global Adherence Scale for Acute Conditions
HBM: Health Belief Model
HCAHPS: Hospital Consumer Assessment of Healthcare Providers
IQR: Interquartile Range
O2sat: Oxygen Saturation
REDCap: Research Electronic Data Capture
RDV: Remdesivir
RPM: Remote Patient Monitoring
SARS-CoV-2: Severe acute respiratory syndrome coronavirus 2
STOFHLA: Short Test of Functional Health Literacy in Adults
T2B: Top 2 Box score
UTAUT: Unified Theory of Acceptance and Use of Technology
