# Supplementary material for: Care at home for remdesivir treatment of COVID-19: a survey study of patient and physician experiences
Source: BMC Infect Dis. 2025 Oct 23;25:1398. doi: 10.1186/s12879-025-11737-1 (PMC12548261; doi:10.1186/s12879-025-11737-1)
Supplement: Supplementary file 1 — Supplementary Material 1: Table 2. Summary of Patient Survey Responses: Hospital Re-admission Indicator [file 12879_2025_11737_MOESM1_ESM.docx]

**Table 2. Summary of Patient Survey Responses: Hospital Re-admission Indicator**

|  | Re-admission_indicator | |  |  |  |  |
| --- | --- | --- | --- | --- | --- | --- |
| **Demographics** | Y (N=60) | N (N=479) | Total (N=539) | | P-value |  |
| **Age** |  |  |  | | 0.0023^1^ |  |
| N | 60 | 479 | 539 | |  |  |
| Mean (SD) | 64.7 (16.67) | 57.6 (16.03) | 58.4 (16.24) | |  |  |
| Median | 66 | 58 | 59 | |  |  |
| Range | 23.0, 96.0 | 18.0, 92.0 | 18.0, 96.0 | |  |  |
|  |  |  |  | |  |  |
| **GENDER**, n (%) |  |  |  | | 0.5935^2^ |  |
| F | 25 (41.7%) | 217 (45.3%) | 242 (44.9%) | |  |  |
| M | 35 (58.3%) | 262 (54.7%) | 297 (55.1%) | |  |  |
|  |  |  |  | |  |  |
| **Race**, n (%) |  |  |  | | 0.1499^2^ |  |
| Asian/PI | 6 (10.0%) | 23 (4.8%) | 29 (5.4%) | |  |  |
| Black | 11 (18.3%) | 57 (11.9%) | 68 (12.6%) | |  |  |
| Hispanic | 23 (38.3%) | 238 (49.7%) | 261 (48.4%) | |  |  |
| White | 20 (33.3%) | 154 (32.2%) | 174 (32.3%) | |  |  |
| Other/Unk | 0 (0.0%) | 7 (1.5%) | 7 (1.3%) | |  |  |
| **Highest grade or level of school completed** n (%) |  |  |  | | 0.4642^2^ |  |
| < 12th grade | 4 (6.9%) | 28 (5.9%) | 32 (6.0%) | |  |  |
| High school graduate or GED | 14 (24.1%) | 92 (19.4%) | 106 (19.9%) | |  |  |
| Some college or tech sch | 17 (29.3%) | 200 (42.2%) | 217 (40.8%) | |  |  |
| Completed Bachelor or higher | 21 (36.2%) | 142 (30.0%) | 163 (30.6%) | |  |  |
| Do not want to answer | 2 (3.4%) | 12 (2.5%) | 14 (2.6%) | |  |  |
| Missing | 2 | 5 | 7 | |  |  |
| **In general, how would you rate your overall health?** n (%) |  |  |  | | 0.0778^2^ |  |
| Poor | 7 (11.7%) | 30 6.3%) | 37 (6.9%) | |  |  |
| Fair | 23 (38.3%) | 121 (25.5%) | 144 (26.9%) | |  |  |
| Good | 18 (30.0%) | 179 (37.7%) | 197 (36.8%) | |  |  |
| Very Good | 9 (15.0%) | 103 (21.7%) | 112 (20.9%) | |  |  |
| Excellent | 3 (5.0%) | 42 (8.8%) | 45 (8.4%) | |  |  |
|  |  |  |  | |  |  |
| **How often do you have someone (like a family member, friend, hospital clinic worker, or caregiver) help you read hospital or other medical materials?** n (%) |  |  |  | | 0.8860^2^ |  |
| None of the time | 30 (51.7%) | 268 (56.8%) | 298 (56.2%) | |  |  |
| A little of the time | 6 (10.3%) | 40 (8.5%) | 46 (8.7%) | |  |  |
| Some of the time | 8 (13.8%) | 55 (11.7%) | 63 (11.9%) | |  |  |
| Most of the time | 3 (5.2%) | 33 (7.0%) | 36 (6.8%) | |  |  |
| All of the time | 11 (19.0%) | 76 (16.1%) | 87 (16.4%) | |  |  |
|  |  |  |  | |  |  |
| **How often do you have problems learning about your medical condition because of difficulty understanding written information?** n (%) |  |  |  | | 0.0248^2^ |  |
| None of the time | 30 (50.0%) | 317 (67.0%) | 347 (65.1%) | |  |  |
| A little of the time | 14 (23.3%) | 57 (12.1%) | 71 (13.3%) | |  |  |
| Some of the time | 7 (11.7%) | 56 (11.8%) | 63 (11.8%) | |  |  |
| Most or all of the time | 9 (15.0%) | 43 (9.1%) | 52 (9.8%) | |  |  |
|  | |  |  |  | |  |
| **1. When remdesivir was offered, was adequate information provided?** n (%) | |  |  |  | | 0.1527^2^ |
| Never | 11 (19.3%) | 91 (20.0%) | 102 (19.9%) | |  |  |
| Sometimes | 12 (21.1%) | 52 (11.4%) | 64 (12.5%) | |  |  |
| Usually | 10 (17.5%) | 68 (14.9%) | 78 (15.2%) | |  |  |
| Always | 24 (42.1%) | 244 (53.6%) | 268 (52.3%) | |  |  |
| **2. When remdesivir was offered, how comfortable did it make you feel to know that this medication was Emergency Use Authorized?** N (%) |  |  |  | | 0.1021^2^ |  |
| Not comfortable | 10 (17.5%) | 52 (11.6%) | 62 (12.3%) | |  |  |
| Somewhat comfortable | 13 (22.8%) | 98 (21.9%) | 111 (22.0%) | |  |  |
| Comfortable | 25 (43.9%) | 160 (35.8%) | 185 (36.7%) | |  |  |
| Very Comfortable | 9 (15.8%) | 137 (30.6%) | 146 (29.0%) | |  |  |
| **3. How comfortable did you feel that the benefits of remdesivir treatment outweighed any potential risks?** n (%) |  |  |  | | 0.0327^2^ |  |
| Not comfortable | 9 (15.0%) | 35 (7.6%) | 44 (8.5%) | |  |  |
| Somewhat comfortable | 18 (30.0%) | 103 (22.5%) | 121 (23.4%) | |  |  |
| Comfortable | 24 (40.0%) | 184 (40.2%) | 208 (40.2%) | |  |  |
| Very Comfortable | 9 (15.0%) | 136 (29.7%) | 145 (28.0%) | |  |  |
| **4. After remdesivir treatment for COVID-19, did it relieve concerns about COVID-19 infection leading to disability?** n (%) |  |  |  | | 0.6381^2^ |  |
| Yes | 26 (44.1%) | 220 (47.3%) | 246 (46.9%) | |  |  |
| No | 33 (55.9%) | 245 (52.7%) | 278 (53.1%) | |  |  |
| **5. After remdesivir treatment did you feel worried about the likelihood of spreading COVID-19infection to others?** n (%) |  |  |  | | 0.4512^2^ |  |
| Yes | 12 (20.7%) | 120 (25.2%) | 132 (24.7%) | |  |  |
| No | 46 (79.3%) | 356 (74.8%) | 402 (75.3%) | |  |  |
| **6. After remdesivir treatment did you feel worried about COVID-19infection complications?** n (%) |  |  |  | | 0.1347^2^ |  |
| Yes | 29 (49.2%) | 185 (39.0%) | 214 (40.2%) | |  |  |
| No | 30 (50.8%) | 289 (61.0%) | 319 (59.8%) | |  |  |
| Missing | 1 | 5 | 6 | |  |  |
| Never | 11 (20.0%) | 54 (12.4%) | 65 (13.2%) | |  |  |
| Sometimes | 8 (14.5%) | 41 (9.4%) | 49 (10.0%) | |  |  |
| Usually | 8 (14.5%) | 63 (14.4%) | 71 (14.5%) | |  |  |
| Always | 28 (50.9%) | 278 (63.8%) | 306 (62.3%) | |  |  |
| **7. Before giving you any new medicine, how often did hospital staff describe possible side effects in a way you could understand?** n (%) |  |  |  | | 0.5751^2^ |  |
| Never | 21 (35.0%) | 152 (33.6%) | 173 (33.8%) | |  |  |
| Sometimes | 12 (20.0%) | 85 (18.8%) | 97 (18.9%) | |  |  |
| Usually | 12 (20.0%) | 67 (14.8%) | 79 (15.4%) | |  |  |
| Always | 15 (25.0%) | 148 (32.7%) | 163 (31.8%) | |  |  |
| **8. After you left the hospital, did you go directly to your own home, to someone else’s home, or to another health facility?** n (%) |  |  |  | | 0.4872^2^ |  |
| Directly to my own home | 56 (93.3%) | 454 (95.4%) | 510 (95.1%) | |  |  |
| Other | 4 (6.7%) | 22 (4.6%) | 26 (4.9%) | |  |  |
| **9. After you left the hospital, did you have someone to help you to take care of daily needs?** n (%) |  |  |  | | 0.4671^2^ |  |
| None of the time | 4 (6.8%) | 46 (9.7%) | 50 (9.4%) | |  |  |
| A little of the time | 2 (3.4%) | 36 (7.6%) | 38 (7.1%) | |  |  |
| Some of the time | 8 (13.6%) | 74 (15.6%) | 82 (15.4%) | |  |  |
| All of the time | 45 (76.3%) | 319 (67.2%) | 364 (68.2%) | |  |  |
| **10. After you left the hospital, did you have someone to take you to the doctor if you needed it?** n (%) |  |  |  | | 0.6687^2^ |  |
| None of the time | 5 (8.3%) | 57 (12.0%) | 62 (11.6%) | |  |  |
| A little of the time | 2 (3.3%) | 20 (4.2%) | 22 (4.1%) | |  |  |
| Some of the time | 4 (6.7%) | 45 (9.5%) | 49 (9.2%) | |  |  |
| All of the time | 49 (81.7%) | 353 (74.3%) | 402 (75.1%) | |  |  |
| **11. After you left the hospital, did doctors, nurses or other hospital staff talk with you about whether you would have the help you needed when you left the hospital?** n (%) |  |  |  | | 0.2653^2^ |  |
| Never | 10 (17.2%) | 60 (13.1%) | 70 (13.6%) | |  |  |
| Sometimes | 8 (13.8%) | 43 (9.4%) | 51 (9.9%) | |  |  |
| Usually | 10 (17.2%) | 57 (12.5%) | 67 (13.0%) | |  |  |
| Always | 30 (51.7%) | 297 (65.0%) | 327 (63.5%) | |  |  |
| **12. Did you get information inwriting about what symptoms or health problems to look out for after you left the hospital?** n (%) |  |  |  | | 0.1929^2^ |  |
| Never | 11 (20.0%) | 54 (12.4%) | 65 (13.2%) | |  |  |
| Sometimes | 8 (14.5%) | 41 (9.4%) | 49 (10.0%) | |  |  |
| Usually | 8 (14.5%) | 63 (14.4%) | 71 (14.5%) | |  |  |
| Always | 28 (50.9%) | 278 (63.8%) | 306 (62.3%) | |  |  |
| **13. How prepared were you to follow home isolation guidelines while receiving remdesivir treatment?** n (%) |  |  |  | | 0.1774^2^ |  |
| Not prepared | 3 (5.0%) | 37 (7.9%) | 40 (7.5%) | |  |  |
| Somewhat prepared | 20 (33.3%) | 107 (22.8%) | 127 (24.0%) | |  |  |
| Very prepared | 21 (35.0%) | 150 (31.9%) | 171 (32.3%) | |  |  |
| Extremely prepared | 16 (26.7%) | 176 (37.4%) | 192 (36.2%) | |  |  |
| **14. How comfortable did you feel to be discharged as an outpatient, with remdesivir treatment forCOVID-19?** n (%) |  |  |  | | 0.3117^2^ |  |
| Not comfortable | 7 (12.1%) | 44 (9.4%) | 51 (9.7%) | |  |  |
| Somewhat comfortable | 12 (20.7%) | 69 (14.7%) | 81 (15.4%) | |  |  |
| Comfortable | 21 (36.2%) | 153 (32.7%) | 174 (33.1%) | |  |  |
| Very Comfortable | 18 (31.0%) | 202 (43.2%) | 220 (41.8%) | |  |  |
| **15. How easy would it have been to return to the medical center for daily treatment if your condition worsened?** n (%) |  |  |  | | 0.0704^2^ |  |
| Very difficult | 10 (16.9%) | 54 (11.4%) | 64 (12.0%) | |  |  |
| Somewhat difficult | 8 (13.6%) | 94 (19.9%) | 102 (19.2%) | |  |  |
| Somewhat easy | 19 (32.2%) | 97 (20.5%) | 116 (21.8%) | |  |  |
| Very easy | 22 (37.3%) | 228 (48.2%) | 250 (47.0%) | |  |  |
| **16. How satisfied were you with the nursing care provided during your remdesivir treatment as an outpatient?** n (%) |  |  |  | | <.0001^2^ |  |
| Very or somewhat dissatisfied | 7 (12.3%) | 20 (4.2%) | 27 (5.1%) | |  |  |
| Somewhat satisfied | 15 (26.3%) | 47 (10.0%) | 62 (11.7%) | |  |  |
| Very satisfied | 35 (61.4%) | 405 (85.8%) | 440 (83.2%) | |  |  |
| **17. If you were asked to input data about your symptoms into a smartphone application, how easy was it for you to do?** n (%) |  |  |  | | 0.0834^2^ |  |
| Very or somewhat difficult | 4 (6.9%) | 26 (5.5%) | 30 (5.6%) | |  |  |
| Somewhat easy | 6 (10.3%) | 48 (10.1%) | 54 (10.2%) | |  |  |
| Very easy | 8 (13.8%) | 140 (29.6%) | 148 (27.9%) | |  |  |
| Do not recall inputting data about my symptoms into a smartphone application | 40 (69.0%) | 259 (54.8%) | 299 (56.3%) | |  |  |
|  |  |  |  | |  |  |
| **18. Did you receive medical follow-up at home with an in-person visit or via remote tele-health visit with a care team member?** n (%) |  |  |  | | 0.0012^2^ |  |
| Unverified | 36 (61.0%) | 381 (79.9%) | 417 (77.8%) | |  |  |
| Yes | 9 (15.3%) | 52 (10.9%) | 61 (11.4%) | |  |  |
| 3 | 14 (23.7%) | 44 (9.2%) | 58 (10.8%) | |  |  |
| **19. How satisfied were you with medical follow-up you received?** N (%) |  |  |  | | 0.0071^2^ |  |
| Very or somewhat dissatisfied | 2 (9.1%) | 14 (4.8%) | 16 (5.1%) | |  |  |
| Somewhat satisfied | 8 (36.4%) | 39 (13.4%) | 47 (15.0%) | |  |  |
| Very satisfied | 12 (54.5%) | 239 (81.8%) | 251 (79.9%) | |  |  |
| **20. If you had a telehealth visit, how would you rate difficulty in setting up the telemedicine platform for the visit?** n (%) |  |  |  | | 0.1885^2^ |  |
| Very or somewhat difficult | 0 (0.0%) | 21 (7.2%) | 21 (6.7%) | |  |  |
| Somewhat easy | 2 (9.5%) | 31 (10.6%) | 33 (10.5%) | |  |  |
| Very easy | 6 (28.6%) | 124 (42.5%) | 130 (41.5%) | |  |  |
| No tele-health visit | 13 (61.9%) | 116 (39.7%) | 129 (41.2%) | |  |  |
| **21. How satisfied were you with your tele-health visit?** n (%) |  |  |  | | 0.0992^2^ |  |
| Very or somewhat dissatisfied | 1 (14.3%) | 6 (3.8%) | 7 (4.2%) | |  |  |
| Somewhat satisfied | 3 (42.9%) | 31 (19.4%) | 34 (20.4%) | |  |  |
| Very satisfied | 3 (42.9%) | 123 (76.9%) | 126 (75.4%) | |  |  |
| Missing | 53 | 319 | 372 | |  |  |
|  |  |  |  | |  |  |
| **22. If you had additional questions about remdesivir, how easy was it for you to obtain medication information from your providers?** n (%) |  |  |  | | 0.2888^2^ |  |
| Very or somewhat difficult | 11 (19.0%) | 54 (11.4%) | 65 (12.2%) | |  |  |
| Somewhat easy | 8 (13.8%) | 61 (12.9%) | 69 (13.0%) | |  |  |
| Very easy | 21 (36.2%) | 165 (34.8%) | 186 (35.0%) | |  |  |
| Have no questions | 18 (31.0%) | 194 (40.9%) | 212 (39.8%) | |  |  |
| **23. Do you agree that remdesivir treatment decreased duration of your COVID-19 infection or reduced complications?** n (%) |  |  |  | | 0.0228^2^ |  |
| Disagree or somewhat disagree | 8 (14.0%) | 39 (8.2%) | 47 (8.8%) | |  |  |
| Neutral | 17 (29.8%) | 109 (22.9%) | 126 (23.6%) | |  |  |
| Somewhat Agree | 15 (26.3%) | 86 (18.0%) | 101 (18.9%) | |  |  |
| Strongly Agree | 17 (29.8%) | 243 (50.9%) | 260 (48.7%) | |  |  |
| Missing | 3 | 2 | 5 | |  |  |
|  |  |  |  | |  |  |
| **24. If you had to pay for remdesivir, how much cost-share(co-pay) would you be willing to pay for this treatment per dose?** N (%) |  |  |  | | 0.1824^2^ |  |
| Would not be willing to pay | 34 (58.6%) | 193 (42.6%) | 227 (44.4%) | |  |  |
| $50 USD | 16 (27.6%) | 161 (35.5%) | 177 (34.6%) | |  |  |
| $100 USD | 4 (6.9%) | 39 (8.6%) | 43 (8.4%) | |  |  |
| $200 USD | 2 (3.4%) | 17 (3.8%) | 19 (3.7%) | |  |  |
| $300 USD | 2 (3.4%) | 43 (9.5%) | 45 (8.8%) | |  |  |
| **25. Do you feel that your recovery is the same, better, or worse by receiving remdesivir treatment in the outpatient setting?** n (%) |  |  |  | | 0.0101^2^ |  |
| Much or somewhat worse | 7 (12.3%) | 23 (5.1%) | 30 (5.9%) | |  |  |
| About the same | 21 (36.8%) | 109 (24.3%) | 130 (25.7%) | |  |  |
| Somewhat better | 12 (21.1%) | 99 (22.0%) | 111 (21.9%) | |  |  |
| Much better | 17 (29.8%) | 218 (48.6%) | 235 (46.4%) | |  |  |
| **26. How often did nurses treat you with courtesy and respect?** n (%) |  |  |  | | 0.1580^2^ |  |
| Never or sometimes | 4 (6.8%) | 14 (3.0%) | 18 (3.4%) | |  |  |
| Usually | 6 (10.2%) | 30 (6.4%) | 36 (6.8%) | |  |  |
| Always | 49 (83.1%) | 427 (90.7%) | 476 (89.8%) | |  |  |
| **27. How often did nurses listen carefully to you? n (%)** |  |  |  | | 0.56752 |  |
| Never or sometimes | 3 (5.2%) | 22 (4.7%) | 25 (4.7%) | |  |  |
| Usually | 7 (12.1%) | 38 (8.1%) | 45 (8.5%) | |  |  |
| Always | 48 (82.8%) | 412 (87.3%) | 460 (86.8%) | |  |  |
| **28. How often did nurses explain things in a way you could understand? n (%)** |  |  |  | | 0.00492 |  |
| Never or sometimes | 7 (11.9%) | 36 (7.6%) | 43 (8.1%) | |  |  |
| Usually | 14 (23.7%) | 50 (10.6%) | 64 (12.1%) | |  |  |
| Always | 38 (64.4%) | 386 (81.8%) | 424 (79.8%) | |  |  |
| **29. During your remdesivir treatment, how often did doctors treat you with courtesy and respect? n (%)** |  |  |  | | 0.00102 |  |
| Never or sometimes | 8 (14.0%) | 35 (7.5%) | 43 (8.2%) | |  |  |
| Usually | 13 (22.8%) | 43 (9.2%) | 56 (10.7%) | |  |  |
| Always | 36 (63.2%) | 388 (83.3%) | 424 (81.1%) | |  |  |
| **30. During your remdesivir treatment, how often did doctors listen carefully to you? n (%)** |  |  |  | | 0.00232 |  |
| Never or sometimes | 12 (21.1%) | 46 (9.9%) | 58 (11.1%) | |  |  |
| Usually | 12 (21.1%) | 54 (11.6%) | 66 (12.6%) | |  |  |
| Always | 33 (57.9%) | 366 (78.5%) | 399 (76.3%) | |  |  |
| **31. What factors influenced you the most to accept treatment for your COVID-19 infection? Check all that apply.**  **Friends**, n (%) | 6 (10.0%) | 66 (13.8%) | 72 (13.4%) | | 0.4173^2^ |  |
| **Family**, n (%) | 26 (43.3%) | 181 (37.8%) | 207 (38.4%) | | 0.4050^2^ |  |
| **CDC guidelines**, n (%) | 5 (8.3%) | 85 (17.7%) | 90 (16.7%) | | 0.0654^2^ |  |
| **Social Media**, n (%) | 1 (1.7%) | 33 (6.9%) | 34 (6.3%) | | 0.1167^2^ |  |
| **Internet**, n (%) | 2 (3.3%) | 37 (7.7%) | 39 (7.2%) | | 0.2159^2^ |  |
| **Physicians**, n (%) | 44 (73.3%) | 337 (70.4%) | 381 (70.7%) | | 0.6328^2^ |  |
| **News broadcasting**, n (%) | 5 (8.3%) | 50 (10.4%) | 55 (10.2%) | | 0.6116 |  |

^1^Wilcoxon Rank Sum p-value; ^2^Chi-Square p-value
