# Supplementary material for: Care at home for remdesivir treatment of COVID-19: a survey study of patient and physician experiences
Source: BMC Infect Dis. 2025 Oct 23;25:1398. doi: 10.1186/s12879-025-11737-1 (PMC12548261; doi:10.1186/s12879-025-11737-1)
Supplement: Supplementary file 2 — Supplementary Material 2: Table 3. Summary of Physician Survey Responses [file 12879_2025_11737_MOESM2_ESM.docx]

**Table 3. Summary of Physician Survey Responses**

|  | **12. I feel comfortable ordering new or unfamiliar medications that are under emergency use authorization.** | | |  | |
| --- | --- | --- | --- | --- | --- |
|  | Disagree (N=34) | Agree (N=88) | Strongly Agree (N=16) | Total (N=138) | P-value |
| 1. **Where do you work?**   **Emergency medicine**, n (%) |  |  |  |  | 0.1909^1^ |
|  | 6 (17.6%) | 12 (13.6%) | 0 (0.0%) | 18 (13.0%) |  |
|  |  |  |  |  |  |
| **Hospital**, n (%) |  |  |  |  | 0.5807^2^ |
|  | 24 (70.6%) | 60 (68.2%) | 9 (56.3%) | 93 (67.4%) |  |
|  |  |  |  |  |  |
| **Continuing Care**, n (%) |  |  |  |  | 0.9231^1^ |
|  | 3 (8.8%) | 10 (11.4%) | 2 (12.5%) | 15 (10.9%) |  |
|  |  |  |  |  |  |
| **Acute Medical Care at Home**, n (%) |  |  |  |  | 0.1966^1^ |
|  | 3 (8.8%) | 2 (2.3%) | 0 (0.0%) | 5 (3.6%) |  |
|  |  |  |  |  |  |
| **Care Without Delay**, n (%) |  |  |  |  | 0.6417^1^ |
|  | 3 (8.8%) | 5 (5.7%) | 0 (0.0%) | 8 (5.8%) |  |
|  |  |  |  |  |  |
| **Administration e.g. Medical Director**, n (%) |  |  |  |  | 0.9999^1^ |
|  | 0 (0.0%) | 1 (1.1%) | 0 (0.0%) | 1 (0.7%) |  |
|  |  |  |  |  |  |
| **Infectious Disease**, n (%) |  |  |  |  | 0.04^1^ |
|  | 1 (2.9%) | 2 (2.3%) | 3 (18.8%) | 6 (4.3%) |  |
|  |  |  |  |  |  |
| **Other**, n (%) |  |  |  |  | 0.1500^1^ |
|  | 1 (2.9%) | 9 (10.2%) | 3 (18.8%) | 13 (9.4%) |  |
|  |  |  |  |  |  |
| 1. **How many years have you…**   **…been in your current position?** n (%) |  |  |  |  | 0.5468^1^ |
| 1-10yrs | 19 (55.9%) | 38 (43.2%) | 10 (62.5%) | 67 (48.6%) |  |
| 11-20yrs | 11 (32.4%) | 36 (40.9%) | 4 (25.0%) | 51 (37.0%) |  |
| 21-26+yrs | 4 (11.8%) | 14 (15.9%) | 2 (12.5%) | 20 (14.5%) |  |
|  |  |  |  |  |  |
| **…actively been practicing medicine?** n (%) |  |  |  |  | 0.4133^2^ |
| 1-10yrs | 21 (61.8%) | 38 (43.2%) | 8 (50.0%) | 67 (48.6%) |  |
| 11-20yrs | 10 (29.4%) | 33 (37.5%) | 5 (31.3%) | 48 (34.8%) |  |
| 21-26+yrs | 3 (8.8%) | 17 (19.3%) | 3 (18.8%) | 23 (16.7%) |  |
|  |  |  |  |  |  |
| **4. I feel comfortable with off-label use of COVID-19 treatment based on limited clinical data.** n (%) |  |  |  |  | 0.0245^1^ |
| Disagree | 6 (17.6%) | 7 (8.0%) | 0 (0.0%) | 13 (9.4%) |  |
| Agree | 23 (67.6%) | 54 (61.4%) | 7 (43.8%) | 84 (60.9%) |  |
| Strongly Agree | 5 (14.7%) | 27 (30.7%) | 9 (56.3%) | 41 (29.7%) |  |
|  |  |  |  |  |  |
| **5. A clear plan was communicated regarding policies, procedures, and related work-flow for crisis standards of COVID-19 care.** n (%) |  |  |  |  | 0.9319^1^ |
| Disagree | 3 (8.8%) | 11 (12.5%) | 2 (12.5%) | 16 (11.6%) |  |
| Agree | 19 (55.9%) | 45 (51.1%) | 7 (43.8%) | 71 (51.4%) |  |
| Strongly Agree | 12 (35.3%) | 32 (36.4%) | 7 (43.8%) | 51 (37.0%) |  |
|  |  |  |  |  |  |
| **6. I felt adequately supported by organizational infrastructure to perform my job.** n (%) |  |  |  |  | 0.1458^1^ |
| Disagree | 2 (5.9%) | 16 (18.2%) | 3 (18.8%) | 21 (15.2%) |  |
| Agree | 19 (55.9%) | 43 (48.9%) | 4 (25.0%) | 66 (47.8%) |  |
| Strongly Agree | 13 (38.2%) | 29 (33.0%) | 9 (56.3%) | 51 (37.0%) |  |
|  |  |  |  |  |  |
| **7. The greater the support from leadership, the more likely I will adhere to treatment guidelines.** n(%) |  |  |  |  | 0.2606^1^ |
| Disagree | 1 (2.9%) | 4 (4.5%) | 0 (0.0%) | 5 (3.6%) |  |
| Agree | 20 (58.8%) | 37 (42.0%) | 5 (31.3%) | 62 (44.9%) |  |
| Strongly Agree | 13 (38.2%) | 47 (53.4%) | 11 (68.8%) | 71 (51.4%) |  |
|  |  |  |  |  |  |
| **8. I felt knowledgeable when ordering remdesivir.** n (%) |  |  |  |  | 0.0003^1^ |
| Disagree | 2 (5.9%) | 5 (5.7%) | 0 (0.0%) | 7 (5.1%) |  |
| Agree | 26 (76.5%) | 40 (45.5%) | 3 (18.8%) | 69 (50.0%) |  |
| Strongly Agree | 6 (17.6%) | 43 (48.9%) | 13 (81.3%) | 62 (44.9%) |  |
|  |  |  |  |  |  |
| **9. The workflow to order outpatient remdesivir was easily incorporated into my daily job duties.** n (%) |  |  |  |  | 0.1381^2^ |
| Disagree | 12 (35.3%) | 23 (26.7%) | 3 (18.8%) | 38 (27.9%) |  |
| Agree | 17 (50.0%) | 42 (48.8%) | 5 (31.3%) | 64 (47.1%) |  |
| Strongly Agree | 5 (14.7%) | 21 (24.4%) | 8 (50.0%) | 34 (25.0%) |  |
| Missing | 0 | 2 | 0 | 2 |  |
|  |  |  |  |  |  |
| **10. While I was able to follow organization recommendations for use of COVID-19 medications under emergency use authorization, I still felt there were significant barriers.** n (%) |  |  |  |  | 0.1005^1^ |
| Disagree | 17 (53.1%) | 61 (70.1%) | 8 (50.0%) | 86 (63.7%) |  |
| Agree | 13 (40.6%) | 17 (19.5%) | 5 (31.3%) | 35 (25.9%) |  |
| Strongly Agree | 2 (6.3%) | 9 (10.3%) | 3 (18.8%) | 14 (10.4%) |  |
| Missing | 2 | 1 | 0 | 3 |  |
|  |  |  |  |  |  |
| **11. Actions of my colleagues/peers influenced my decision to adhere to COVID-19 treatment guidelines.** n (%) |  |  |  |  | 0.0042^1^ |
| Disagree | 14 (41.2%) | 32 (36.4%) | 6 (37.5%) | 52 (37.7%) |  |
| Agree | 20 (58.8%) | 46 (52.3%) | 4 (25.0%) | 70 (50.7%) |  |
| Strongly Agree | 0 (0.0%) | 10 (11.4%) | 6 (37.5%) | 16 (11.6%) |  |
|  |  |  |  |  |  |
| **13. Ease of ordering new medications was limited by use of multiple medication ordering platforms within HealthConnect.** n (%) |  |  |  |  | 0.4117^1^ |
| Disagree | 18 (52.9%) | 53 (60.2%) | 10 (62.5%) | 81 (58.7%) |  |
| Agree | 14 (41.2%) | 26 (29.5%) | 3 (18.8%) | 43 (31.2%) |  |
| Strongly Agree | 2 (5.9%) | 9 (10.2%) | 3 (18.8%) | 14 (10.1%) |  |
|  |  |  |  |  |  |
| **14. How confident were you in the effectiveness of remdesivir from Dec 2020- June 2021 associated with alpha, beta, gamma variants?** n (%) |  |  |  |  | 0.0159^1^ |
| Not confident | 3 (9.1%) | 11 (12.5%) | 2 (12.5%) | 16 (11.7%) |  |
| Somewhat Confident | 16 (48.5%) | 35 (39.8%) | 2 (12.5%) | 53 (38.7%) |  |
| Confident | 11 (33.3%) | 38 (43.2%) | 7 (43.8%) | 56 (40.9%) |  |
| Very confident | 3 (9.1%) | 4 (4.5%) | 5 (31.3%) | 12 (8.8%) |  |
|  |  |  |  |  |  |
| **15. How confident were you in the effectiveness of remdesivir from July 2021-Jan 2022 associated with delta variant?** n (%) |  |  |  |  | 0.1995^1^ |
| Not confident | 4 (11.8%) | 11 (12.6%) | 2 (12.5%) | 17 (12.4%) |  |
| Somewhat Confident | 19 (55.9%) | 34 (39.1%) | 4 (25.0%) | 57 (41.6%) |  |
| Confident | 8 (23.5%) | 35 (40.2%) | 6 (37.5%) | 49 (35.8%) |  |
| Very confident | 3 (8.8%) | 7 (8.0%) | 4 (25.0%) | 14 (10.2%) |  |
|  |  |  |  |  |  |
| **16. How confident were you in the effectiveness of remdesivir from Feb 2022 onwards associated with omicron variant?** n (%) |  |  |  |  | 0.2937^1^ |
| Not confident | 2 (6.7%) | 19 (21.6%) | 2 (13.3%) | 23 (17.3%) |  |
| Somewhat Confident | 16 (53.3%) | 33 (37.5%) | 4 (26.7%) | 53 (39.8%) |  |
| Confident | 9 (30.0%) | 30 (34.1%) | 7 (46.7%) | 46 (34.6%) |  |
| Very confident | 3 (10.0%) | 6 (6.8%) | 2 (13.3%) | 11 (8.3%) |  |
|  |  |  |  |  |  |
| **17. Communication was a barrier to order services (e.g., nursing, oxygen via durable medical equipment, patient monitoring kits, monitoring) related to new medications.** n (%) |  |  |  |  | 0.0833^1^ |
| Disagree | 18 (52.9%) | 63 (71.6%) | 8 (53.3%) | 89 (65.0%) |  |
| Agree | 14 (41.2%) | 19 (21.6%) | 4 (26.7%) | 37 (27.0%) |  |
| Strongly Agree | 2 (5.9%) | 6 (6.8%) | 3 (20.0%) | 11 (8.0%) |  |
|  |  |  |  |  |  |
| **18. On a scale of 1-5, how often do/are you…**  **…agree with others,** n(%) |  |  |  |  | 0.5044^1^ |
| 3 | 13 (39.4%) | 42 (48.3%) | 7 (46.7%) | 62 (45.9%) |  |
| 4 | 20 (60.6%) | 42 (48.3%) | 7 (46.7%) | 69 (51.1%) |  |
| 5 Always | 0 (0.0%) | 3 (3.4%) | 1 (6.7%) | 4 (3.0%) |  |
|  |  |  |  |  |  |
| **…consider yourself an extravert,** n (%) |  |  |  |  | 0.1243^1^ |
| 1 Never | 1 (3.0%) | 18 (20.9%) | 2 (12.5%) | 21 (15.6%) |  |
| 2 | 10 (30.3%) | 34 (39.5%) | 4 (25.0%) | 48 (35.6%) |  |
| 3 | 13 (39.4%) | 16 (18.6%) | 6 (37.5%) | 35 (25.9%) |  |
| 4 | 7 (21.2%) | 12 (14.0%) | 2 (12.5%) | 21 (15.6%) |  |
| 5 Always | 2 (6.1%) | 6 (7.0%) | 2 (12.5%) | 10 (7.4%) |  |
|  |  |  |  |  |  |
| **…open to the ideas of others**, n (%) |  |  |  |  | 0.0405^1^ |
| 2-3 | 2 (5.9%) | 17 (19.8%) | 0 (0.0%) | 19 (14.0%) |  |
| 4 | 22 (64.7%) | 45 (52.3%) | 7 (43.8%) | 74 (54.4%) |  |
| 5 Always | 10 (29.4%) | 24 (27.9%) | 9 (56.3%) | 43 (31.6%) |  |
|  |  |  |  |  |  |
| **…conscientious**, n (%) |  |  |  |  | 0.8912^1^ |
| 2-3 | 2 (5.9%) | 5 (5.8%) | 0 (0.0%) | 7 (5.2%) |  |
| 4 | 13 (38.2%) | 38 (44.2%) | 6 (37.5%) | 57 (41.9%) |  |
| 5 Always | 19 (55.9%) | 43 (50.0%) | 10 (62.5%) | 72 (52.9%) |  |
|  |  |  |  |  |  |
| **19. I feel that providing medications like outpatient remdesivir reduced community transmission of COVID-19.** n (%) |  |  |  |  | 0.0286^1^ |
| Disagree | 10 (29.4%) | 26 (30.2%) | 0 (0.0%) | 36 (26.7%) |  |
| Agree | 17 (50.0%) | 42 (48.8%) | 7 (46.7%) | 66 (48.9%) |  |
| Strongly Agree | 7 (20.6%) | 18 (20.9%) | 8 (53.3%) | 33 (24.4%) |  |
|  |  |  |  |  |  |
| **20. New technology products significantly helped with delivery of care to patients.** n (%) |  |  |  |  | 0.6640^1^ |
| Disagree | 3 (8.8%) | 9 (10.6%) | 2 (12.5%) | 14 (10.4%) |  |
| Agree | 24 (70.6%) | 56 (65.9%) | 8 (50.0%) | 88 (65.2%) |  |
| Strongly Agree | 7 (20.6%) | 20 (23.5%) | 6 (37.5%) | 33 (24.4%) |  |
|  |  |  |  |  |  |
| **21. I am confident in my abilities to order and document appropriate administration of remdesivir.** n (%) |  |  |  |  | 0.2556^1^ |
| Disagree | 3 (8.8%) | 7 (8.0%) | 0 (0.0%) | 10 (7.2%) |  |
| Agree | 21 (61.8%) | 49 (55.7%) | 6 (37.5%) | 76 (55.1%) |  |
| Strongly Agree | 10 (29.4%) | 32 (36.4%) | 10 (62.5%) | 52 (37.7%) |  |
|  |  |  |  |  |  |
| **22. The pressure from competitors (e.g. other HealthCare plans or systems) in healthcare is likely to drive my decision on how I practice and treat COVID-19.** n (%) |  |  |  |  | 0.2745^1^ |
| Disagree | 28 (82.4%) | 75 (85.2%) | 15 (93.8%) | 118 (85.5%) |  |
| Agree | 6 (17.6%) | 10 (11.4%) | 0 (0.0%) | 16 (11.6%) |  |
| Strongly Agree | 0 (0.0%) | 3 (3.4%) | 1 (6.3%) | 4 (2.9%) |  |
|  |  |  |  |  |  |
| **23. I feel that infusion treatment with remdesivir in the home relieved utilization of hospital resources.** n (%) |  |  |  |  | 0.3148^1^ |
| Disagree | 3 (8.8%) | 5 (5.7%) | 0 (0.0%) | 8 (5.9%) |  |
| Agree | 15 (44.1%) | 34 (39.1%) | 3 (20.0%) | 52 (38.2%) |  |
| Strongly Agree | 16 (47.1%) | 48 (55.2%) | 12 (80.0%) | 76 (55.9%) |  |
|  |  |  |  |  |  |
| **24. I am confident that there were adequate resources to provide remdesivir infusions.** n (%) |  |  |  |  | 0.0067^1^ |
| Disagree | 17 (50.0%) | 26 (30.2%) | 4 (26.7%) | 47 (34.8%) |  |
| Agree | 15 (44.1%) | 47 (54.7%) | 4 (26.7%) | 66 (48.9%) |  |
| Strongly Agree | 2 (5.9%) | 13 (15.1%) | 7 (46.7%) | 22 (16.3%) |  |
|  |  |  |  |  |  |
| **25. When patients were discharged to home with remdesivir treatment, I feel that there was adequate support for follow-up care through telehealth.** n (%) |  |  |  |  | 0.3869^1^ |
| Disagree | 5 (15.2%) | 14 (16.3%) | 2 (13.3%) | 21 (15.7%) |  |
| Agree | 21 (63.6%) | 53 (61.6%) | 6 (40.0%) | 80 (59.7%) |  |
| Strongly Agree | 7 (21.2%) | 19 (22.1%) | 7 (46.7%) | 33 (24.6%) |  |
|  |  |  |  |  |  |
| **26. I am confident in the abilities of nursing to provide care when patients are discharged with remdesivir treatment.** n (%) |  |  |  |  | 0.9473^1^ |
| Disagree | 4 (12.1%) | 11 (12.6%) | 1 (6.7%) | 16 (11.9%) |  |
| Agree | 21 (63.6%) | 55 (63.2%) | 9 (60.0%) | 85 (63.0%) |  |
| Strongly Agree | 8 (24.2%) | 21 (24.1%) | 5 (33.3%) | 34 (25.2%) |  |
| Missing | 1 | 1 | 1 | 3 |  |
|  |  |  |  |  |  |
| **27. When patients are discharged with remdesivir treatment, I am confident in pharmacy to support the medication administration.** n (%) |  |  |  |  | 0.3492^1^ |
| Disagree | 2 (6.1%) | 3 (3.4%) | 0 (0.0%) | 5 (3.7%) |  |
| Agree | 21 (63.6%) | 50 (57.5%) | 6 (40.0%) | 77 (57.0%) |  |
| Strongly Agree | 10 (30.3%) | 34 (39.1%) | 9 (60.0%) | 53 (39.3%) |  |
|  |  |  |  |  |  |
| **28. I feel confident that the durable medical equipment supplier can deliver adequate supplies to my patients, in a timely manner.** n (%) |  |  |  |  | 0.3362^1^ |
| Disagree | 9 (26.5%) | 18 (20.7%) | 3 (20.0%) | 30 (22.1%) |  |
| Agree | 18 (52.9%) | 57 (65.5%) | 7 (46.7%) | 82 (60.3%) |  |
| Strongly Agree | 7 (20.6%) | 12 (13.8%) | 5 (33.3%) | 24 (17.6%) |  |
|  |  |  |  |  |  |
| **29. I am confident in mechanisms for obtaining labs off-site (e.g. From contracted phlebotomist group).** n (%) |  |  |  |  | 0.3450^1^ |
| Disagree | 7 (21.2%) | 20 (23.5%) | 2 (13.3%) | 29 (21.8%) |  |
| Agree | 22 (66.7%) | 55 (64.7%) | 8 (53.3%) | 85 (63.9%) |  |
| Strongly Agree | 4 (12.1%) | 10 (11.8%) | 5 (33.3%) | 19 (14.3%) |  |
|  |  |  |  |  |  |
| **30. Compared to this time last year, would you say each of the following aspects of your health has gotten better, worse, or stayed about the same?**  **…Overall health**, n (%) |  |  |  |  | 0.9680^1^ |
| Worse | 5 (14.7%) | 15 (17.2%) | 2 (12.5%) | 22 (16.1%) |  |
| Same | 17 (50.0%) | 46 (52.9%) | 9 (56.3%) | 72 (52.6%) |  |
| Better | 12 (35.3%) | 26 (29.9%) | 5 (31.3%) | 43 (31.4%) |  |
| Missing | 0 | 1 | 0 | 1 |  |
|  |  |  |  |  |  |
| **…Physical health**, n (%) |  |  |  |  | 0.7892^1^ |
| Worse | 5 (14.7%) | 18 (20.7%) | 2 (12.5%) | 25 (18.2%) |  |
| Same | 15 (44.1%) | 42 (48.3%) | 8 (50.0%) | 65 (47.4%) |  |
| Better | 14 (41.2%) | 27 (31.0%) | 6 (37.5%) | 47 (34.3%) |  |
|  |  |  |  |  |  |
| **…Mental health**, n (%) |  |  |  |  | 0.4218^1^ |
| Worse | 5 (15.2%) | 20 (23.3%) | 3 (18.8%) | 28 (20.7%) |  |
| Same | 14 (42.4%) | 45 (52.3%) | 9 (56.3%) | 68 (50.4%) |  |
| Better | 14 (42.4%) | 21 (24.4%) | 4 (25.0%) | 39 (28.9%) |  |
| Missing | 1 | 2 | 0 | 3 |  |
|  |  |  |  |  |  |
| **…Anxiety/stress level**, n (%) |  |  |  |  | 0.4209^1^ |
| Worse | 8 (23.5%) | 25 (28.7%) | 4 (26.7%) | 37 (27.2%) |  |
| Same | 13 (38.2%) | 44 (50.6%) | 7 (46.7%) | 64 (47.1%) |  |
| Better | 13 (38.2%) | 18 (20.7%) | 4 (26.7%) | 35 (25.7%) |  |
|  |  |  |  |  |  |
| **31. I felt adequately trained and prepared to provide care during the pandemic.** n (%) |  |  |  |  | 0.3314^1^ |
| Disagree | 11 (33.3%) | 17 (19.5%) | 2 (12.5%) | 30 (22.1%) |  |
| Agree | 18 (54.5%) | 58 (66.7%) | 10 (62.5%) | 86 (63.2%) |  |
| Strongly Agree | 4 (12.1%) | 12 (13.8%) | 4 (25.0%) | 20 (14.7%) |  |
|  |  |  |  |  |  |
| **32. I felt supported by colleague sand medical center leadership tore-allocate resources for treatment of COVID-19 in the outpatient setting.** n (%) |  |  |  |  | 0.0541^1^ |
| Disagree | 5 (15.2%) | 14 (16.5%) | 1 (7.1%) | 20 (15.2%) |  |
| Agree | 22 (66.7%) | 58 (68.2%) | 6 (42.9%) | 86 (65.2%) |  |
| Strongly Agree | 6 (18.2%) | 13 (15.3%) | 7 (50.0%) | 26 (19.7%) |  |
|  |  |  |  |  |  |
| **33. Looking back at the pandemic and your individual/organization performance, if another medica crisis arose, what do you think should be the highest priority to invest to overcome some of the barriers/challenges. (Select one response)** n (%) |  |  |  |  | 0.0160^2^ |
| Technology | 1 (2.9%) | 1 (1.1%) | 1 (6.3%) | 3 (2.2%) |  |
| More labor - physicians, nurses | 17 (50.0%) | 35 (39.8%) | 5 (31.3%) | 57 (41.3%) |  |
| Better training | 2 (5.9%) | 4 (4.5%) | 0 (0.0%) | 6 (4.3%) |  |
| Clear communication | 2 (5.9%) | 11 (12.5%) | 0 (0.0%) | 13 (9.4%) |  |
| Operational infrastructure | 3 (8.8%) | 17 (19.3%) | 3 (18.8%) | 23 (16.7%) |  |
| Leadership | 0 (0.0%) | 6 (6.8%) | 0 (0.0%) | 6 (4.3%) |  |
| Adequate medical supplies/drugs | 9 (26.5%) | 10 (11.4%) | 3 (18.8%) | 22 (15.9%) |  |
| Other | 0 (0.0%) | 4 (4.5%) | 4 (25.0%) | 8 (5.8%) |  |
